# Simultaneous determination of ionic polymers and heavy metal ions concentrations in aqueous solution after their adsorptive removal using eco-friendly activated biocarbons

**DOI:** 10.3389/fchem.2025.1621297

**Published:** 2025-07-02

**Authors:** Marlena Groszek, Małgorzata Wiśniewska, Piotr Nowicki

**Affiliations:** ^1^ Department of Radiochemistry and Environmental Chemistry, Institute of Chemical Sciences, Faculty of Chemistry, Maria Curie-Sklodowska University in Lublin, Lublin, Poland; ^2^ Department of Applied Chemistry, Faculty of Chemistry, Adam Mickiewicz University in Poznań, Poznań, Poland

**Keywords:** activated biocarbons, eco-friendly synthesis, ionic polymers, heavy metal ions, multicomponent solutions, simultaneous adsorption

## Abstract

Growing contamination of aquatic systems by industrial and domestic pollutants necessitates the development of efficient and sustainable wastewater treatment technologies. Activated biocarbons derived from renewable biomass sources have proven to be promising materials for this purpose thanks to their large specific surface area, well-developed porosity, high content of surface groups and cost-effectiveness. This paper describes the preparation, physicochemical characterization and practical application of carbonaceous adsorbents derived from the nettle and mint herbs residues using an environmentally friendly method–single-stage (direct) physical activation with carbon dioxide. The obtained activated biocarbons were fully characterized in terms of their texture, surface properties, and chemical composition, and then used to remove ionic polymers (poly(acrylic acid) and polyethyleneimine) as well as Cd(II) and As(V) ions from aqueous solutions. The influence of the above-mentioned substances on their mutual adsorption was investigated. The obtained eco-friendly carbonaceous materials are characterized by moderately developed surface area (368–666 m^2^/g) and high content of the surface functional groups (2.19–4.89 mmol/g). The maximum adsorbed amounts of ionic polymers reached the level of about 80 mg/g, while those of heavy metal ions varied in the range of 4–19 mg/g. Competitive adsorption between the polymer chains and heavy metal ions was confirmed. In the binary system containing both types of macromolecules, an increase in the adsorbed amounts of poly(acrylic acid) and polyethyleneimine was observed. In turn, the simultaneous presence of ionic polymers and heavy metal ions leads to a reduction in the adsorbed quantities of all adsorbates. The analysis of adsorption-desorption, surface, and electrokinetic data allowed the identification of the most probable mechanisms of separation of ionic polymers and heavy metal ions from the aqueous phase using eco-friendly carbonaceous adsorbents.

## Graphical Abstract

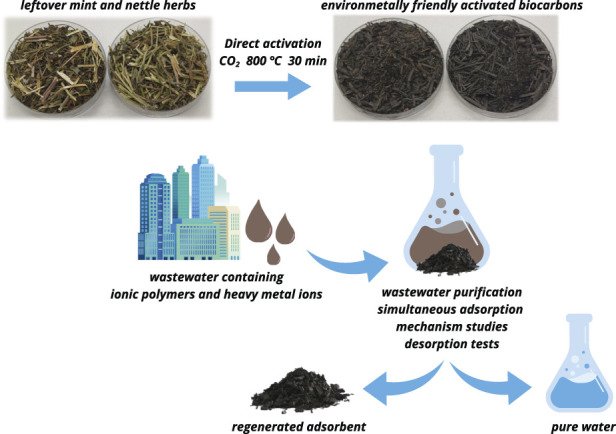

## Introduction

Water is an essential resource for life, but its availability and quality are increasingly threatened by unsustainable consumption and waste management practices (Ali, 2012; Ali and Gupta, 2006). Water purification is the process of removing contaminants, pathogens, and undesirable substances to make it suitable for human consumption, industrial use, or ecological sustainability. Traditional purification methods, such as sedimentation, chemical precipitation, ion exchange, evaporation, filtration, and chlorination have proven effective in providing clean water to millions of people (Ruthiraan et al., 2015). However, the complexity of contemporary water contamination–ranging from microplastics and emerging pollutants to heavy metal ions and pharmaceutical residues–has necessitated the development of advanced purification techniques (Ganesamoorthy et al., 2021; Schwarzenbach et al., 2006; Singh et al., 2018).

In recent years, adsorption has become a highly effective and versatile technique for removing various types of pollutants from aqueous systems (Bhatnagar et al., 2015; Zhou et al., 2015). Among the many adsorption materials available, activated carbons and synthetic resins, have been widely used primarily due to their high adsorption capacities. Activated carbon is a highly porous material with a large surface area, which makes it an excellent adsorbent for removing a broad spectrum of contaminants present in natural water reservoirs (including groundwater runoff from fertilized agricultural fields) as well as municipal and industrial wastewater (Crini and Lichtfouse, 2019; Dias et al., 2007). Its excellent adsorption properties are attributed to hierarchical pore structure, consisting of micropores, mesopores and macropores, as well as the presence of surface functional groups that enable physical and chemical interactions with pollutants (Soonmin and Kabbashi, 2021; Ioannidou, and Zabaniotou, 2007). However, the production of chemically activated carbonaceous materials or synthetic resins often involves high energy consumption, significant carbon footprint, and the generation of potential secondary pollution, raising concerns about their long-term environmental sustainability.

In response to these challenges, there has been growing interest in the development of environmentally friendly adsorbents derived from renewable, biodegradable, and low-cost sources (Shannon et al., 2008). These criteria are fully met by activated biocarbon–green adsorbent obtained from plant precursors via physical activation (Kyzas and Kostoglou, 2014). Compared to chemical variant of activation, which requires the usage of aggressive chemical reagents like orthophosphoric acid or potassium hydroxide, physical activation is environmentally friendly option in this regard. Moreover, post-processing steps related to removal of residual chemicals are eliminated (Yahya et al., 2015; Yang et al., 2010).

In this paper activated biocarbons obtained from the nettle and mint herbs residues (waste from the herbal industry) through simultaneous pyrolysis and physical activation were applied for organic and inorganic pollutants removal from aqueous solutions. Four adsorbates of different ionic character and molecular weight were used in the study, including high-molecular weight anionic poly(acrylic acid) and cationic polyethyleneimine, as well as low-molecular weight cadmium(II) cations and arsenate(V) anions. The selected adsorbates were chosen due to their common presence in wastewater. Poly(acrylic acid) is widely used as a thickening agent, while polyethyleneimine increases the permeability of cell membranes and is extensively used in biotechnology. Heavy metals ions have a negative impact on human health and are released into the environment from many industries processes. The adsorption studies were performed in the solution with three different pH values (i.e. 3, 6 and 9). Due to the greatest adsorbed amounts observed at pH 3, these conditions were chosen for the multicomponent adsorption tests. In order to define the adsorption mechanism, the surface charge density, zeta potential, and aggregate sizes of the solid particles dispersed in aqueous suspensions (containing single and binary adsorbates) were determined.

It should be emphasized that the adsorption of ionic polymers on the surface of activated biocarbons remains a largely underexplored area, and very few studies have addressed their removal from complex, multicomponent systems. This research gap highlights the originality and significance of the presented results. As polymeric contaminants are increasingly detected in various aquatic systems, our findings provide a new and important contribution to the development of more effective remediation strategies for polluted aquatic environments (drinking water, wastewater and natural water reservoirs).

## Experimental

### Materials

Nettle (NE) and mint (MT) herb residues were dried at 110 °C and then their stalks were cut into 1.5–2.0 cm long pieces. Fifteen-gram samples of the precursors were placed in nickel boats and subjected to simultaneous pyrolysis and physical activation in a horizontal resistance furnace equipped with a quartz tube reactor (one-zone model PRW75/LM, Czylok, Jastrzębie-Zdrój, Poland). The process involved annealing the sample at 800 °C for 30 min in a CO_2_ atmosphere with a flow rate of 250 cm^3^/min. After the completion of the one-step activation process, the materials were cooled to room temperature under a nitrogen flow (flow rate of 170 cm^3^/min). The resulting activated products were designated as NE_AP and MT_AP.

Polyethyleneimine (PEI) (Sigma Aldrich, Saint Louis, MO, United States) is a cationic polymer with an average molecular weight of 2000 Da, containing amine groups along its chains. This weak polyelectrolyte has a pK_b_ value at pH 9, at which 50% of its functional groups are dissociated. As the pH decreases, the number of dissociated amine groups increases, leading to the spatial expansion of the polymer chain (Von Harpe et al., 2000).

Poly(acrylic acid) (PAA) (Fluka, Saint Louis, MO, United States) represents the group of anionic polymers and it is characterized by an average molecular weight of 2000 Da. It is also a weak polyelectrolyte and its macromolecules contain carboxyl functional groups, 50% of which dissociate at pH 4.5, to the pK_a_ value of PAA. Below this pH value, the degree of dissociation decreases, leading to the formation of a coiled conformation of the polymer chains. In turn, at pH values above 4.5, the number of dissociated carboxyl groups increases, resulting in a more extended chain conformation (Chibowski et al., 2003).

Cadmium nitrate tetrahydrate (CdN_2_O_6_·4H_2_O, Sigma Aldrich, Saint Louis, United States) was used as a source of Cd(II) cations. This compound dissolves well in water at pH below 8, while above this value, cadmium ions precipitate. As a result, experiments involving Cd(II) were limited to the pH range from 3 to 7.

The As(V) anions used in the study were introduced by potassium dihydrogen arsenate (KH_2_AsO_4_, Sigma Aldrich, Saint Louis, United States). This salt is highly water-soluble over a wide pH range. However, the form of arsenate ions varies with pH changes. At higher pH values, the As(V) ion is fully dehydrated and exists as AsO_4_^3-^ anion. As the pH decreases, the degree of hydration increases, resulting in the occurrence of the following forms: HAsO_4_^2-^, H_2_AsO_4_^−^ and H_3_AsO_4_ (Das and Bezbaruah, 2020).

### Adsorbents characterization

The surface morphology of the activated biocarbons was examined using scanning electron microscopy (SEM) (Quanta 250 FEG, FEI, Waltham, MA, United States). The porosity characteristics, including the total/micropore surface area, total pore/micropore volume, and average pore diameter, were assessed by low-temperature (−196 °C) nitrogen adsorption-desorption measurements performed using an ASAP 2420 apparatus (Micromeritics, Norcross, GA, United States). The functional groups present on the surface of the biocarbons were quantified using the Boehm back titration method (Boehm et al., 1964). In this analytical procedure, NaOH and HCl solutions (both at a concentration of 0.1 mol/dm^3^, Avantor Performance Materials, Gliwice, Poland) were used as titrants. To analyze the surface chemistry and identify C-, N-, and O-containing functional groups present on the tested materials, X-ray photoelectron spectroscopy (XPS) (Gammadata Scienta, Uppsala, Sweden) was applied. The ash content was determined according to the ISO 1171:2002 standard, using a muffle furnace FCF-V70C (Czylok, Jastrzębie Zdrój, Poland). The total content of C, N, H, and S was determined using an elemental analyzer Vario EL III (Elementar Analysen Systeme GmbH, Germany). The chemical composition of the materials was also studied by means of X-ray fluorescence (XRF) spectrometry (Axios mAX, PANanalytical, Almelo, Netherlands).

### Adsorption and desorption studies

The adsorbed amounts of PAA and PEI polymers, as well as Cd(II) and As(V) ions, were determined using the static method. This approach involved measuring the change in adsorbate concentration in the solution before and after the adsorption process.

**FIGURE 1:**
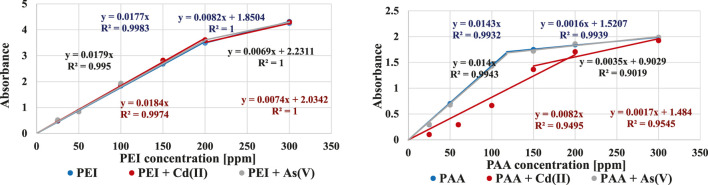
Calibration curves for PEI and PAA in the absence and presence of heavy metal ions (with concentration of 200 ppm).

The concentrations of the polymers were determined using a Carry 100 UV–Vis spectrophotometer (Varian, Palo Alto, Santa Clara, CA, United States). For poly(acrylic acid), its reaction with hyamine 1622 was carried out. As a result, a white-coloured complex, absorbing light at a wavelength of 500 nm, was formed (Crummett and Hummel, 1963). In turn, the concentration of polyethyleneimine was determined based on its reaction with copper(II) chloride, which results in a blue-coloured complex absorbing light at 285 nm (Patkowski et al., 2016). The calibration curves used to determine the polymer concentration determination are presented in Figure 1. The presence of heavy metal ions causes a shift of calibration curves (especially in the case of PAA). These effects were described in details in the previous paper (Gęca et al., 2024c).

The concentrations of Cd(II) and As(V) ions were determined using Inductively Coupled Plasma-Optical Emission Spectrometry (ICP-OES) with the iCAP™ 7200 analyser (Thermo Fisher Scientific, Waltham, United States). The measurement wavelengths were 228.8 nm for cadmium and 189 nm for arsenic ions, respectively. The operational parameters for the ICP-OES included the plasma with the following settings: flow rate of 15 dm^3^/min, a nebulizer flow rate of 0.75 dm^3^/min and a power setting of 1 kW. The presence of the polymers reduced the analytical signal by 5% and this interference was taken into account in the calculations.

Adsorption isotherms were studied over a 24-h period of time. For these tests, 10 cm^3^ of solutions containing the adsorbate (with initial concentrations ranging from 10 to 300 ppm) and a 0.001 mol/dm^3^ NaCl supporting electrolyte were prepared. The weights of activated biocarbons used were 0.02 g for polymers and 0.05 g for metal ions. Experiments were conducted at pH 3, 6 and 9, but studies involving Cd(II) ions were limited to pH 3 and 6. At pH above 7, cadmium(II) ions precipitate in the solution. Therefore, their loss from solution at alkaline pH values is the result not only of Cd(II) ions adsorption but also of precipitation. Before the adsorption test, the pH value of all tested samples was adjusted using 0.1 mol/dm^3^ NaOH or HCl solutions and measured using a Φ360 pH meter (Beckman, Brea, United States). A constant pH level was maintained throughout the adsorption process by continuous monitoring and correction as necessary. The adsorption isotherms data obtained for heavy metal ions were fitted to theoretical models. The Langmuir (Equation 1), Freundlich (Equation 2), Temkin (Equation 3) and Dubinin-Radushkevich (Equation 4) equations were applied (Awad et al., 2019):

$$q_{e}=\frac{q_{m}K_{L}C_{e}}{1+K_{L}C_{e}}$$ (1)

$$q_{e}=K_{F}C_{e}^{1/n}$$ (2)

$$q_{e}=\frac{RT}{b_{T}}\ln A_{T}+\frac{RT}{b_{T}}\ln C_{e}$$ (3)

$$q_{e}=q_{m}\exp\left(-\beta \varepsilon^{2}\right)$$ (4)

where: *q*_*e*_–the adsorbed amount at the equilibrium state [mg/g], *q*_*m*_–the maximum adsorption capacity in the Langmuir model [mg/g], *K*_*L*_–the Langmuir parameter [m^3^/mg], *C*_*e*_–the equilibrium aqueous phase concentration [mg/dm^3^], *K*_*F*_–the Freundlich parameter [mg/g (mg/dm^3^)^1/n^], *n*–the Freundlich constant, *R*–gas constant (8.314 J/(mol K), *T*–temperature [K], *A*_*T*_–Temkin constant [J/mol], *b*_*T*_–Temkin constant [dm^3^/g], *β*–Dubinin-Radushkevich constant, *ε*–adsorption potential [kJ/mol].

Based on the obtained results, pH 3 at which the adsorbed amounts of polymers were the greatest, was chosen for the kinetics studies. In these tests, an appropriate mass of activated biocarbon was added to 10 cm^3^ of a solution containing 0.001 mol/dm^3^ NaCl as the supporting electrolyte. The initial concentration of both polymers was 200 ppm. Adsorption progress was monitored over time intervals ranging from 0.5 to 7 h. Kinetics data for polymers were fitted to the pseudo-first-order (Equation 5) and pseudo-second-order (Equation 6) models (Wang and Guo, 2020), according to the equations:

$$\frac{{dq}_{t}}{dt}=k_{1}\left(q_{e}-q_{t}\right)$$ (5)

$$\frac{{dq}_{t}}{dt}=k_{2}{\left(q_{e}-q_{t}\right)}^{2}$$ (6)

where: *q*_*e*_–the adsorbed amount in the equilibrium state [mg/g], *q*_*t*_–the adsorbed amount after time “t” [mg/g], *k*_*1*_ – the equilibrium rate constant [1/min], *k*_*2*_ – the equilibrium rate constant [g/(mg⋅min)].

Adsorption studies in single and binary adsorbate systems were conducted for 24 h at pH 3. The 10 cm^3^ of the suspensions consisted of: 200 ppm adsorbate (polymers and/or metal ions), 0.001 mol/dm^3^ NaCl and appropriate mass of activated biocarbon, were prepared. After the adsorption process, activated biocarbon was separated using a microcentrifuge (Centrifuge MPW 233e, MPW Med. Instruments, Warsaw, Poland). The concentrations of adsorbates in the supernatants were measured. For polymers, desorption from the surface of biocarbon materials was also investigated, while for metal ions, desorption studies were not conducted due to the small amounts adsorbed and the high associated errors. The H_2_O, HNO_3_ or NaOH solutions (acidic and basic solutions with a concentration of 0.1 mol/dm^3^) were used as desorbing agents, and the process lasted 24 h. After that time activated biocarbon was again separated and the concentrations of PAA or PEI in the supernatants were determined. The NE_AP activated biocarbon was dried after the desorption process and performed to the adsorption-desorption cycle studies. All adsorption and desorption tests were carried out at 25 °C. Each sample was measured 3 times and the differences between the obtained result values do not exceed 5%.

### Colloidal properties of activated biocarbons suspensions without and with adsorbates

The determinations of surface charge density (σ_0_), zeta potential (ζ) and aggregates size were carried out at 25 °C for the following systems: NE_AP, NE_AP + PEI, NE_AP + PAA, NE_AP + PAA + PEI, NE_AP + Cd(II), NE_AP + Cd(II)+PEI, NE_AP + Cd(II)+PAA, NE_AP + As(V), NE_AP + As(V)+PEI, NE_AP + As(V)+PAA, MT_AP, MT_AP + PEI, MT_AP + PAA, MT_AP + PAA + PEI, MT_AP + Cd(II), MT_AP + Cd(II)+PEI, MT_AP + Cd(II)+PAA, MT_AP + As(V), MT_AP + As(V)+PEI and MT_AP + As(V)+PAA.

To determine the surface charge density (σ_0_), 50 cm^3^ of suspensions were prepared, each containing the given adsorbate at an initial concentration of 100 ppm, supporting electrolyte NaCl at a concentration of 0.001 mol/dm^3^ and 0.054 g of NE_AP or 0.03 g of MT_AP. The prepared suspension was placed in a thermostated Teflon vessel (RE 204 thermostat, Lauda Scientific, Lauda-Königshofen, Germany). The glass and calomel electrodes (Beckman Instruments, Brea, United States) were introduced into the vessel to monitor pH changes using a pH-meter (pHM 240, Radiometer, Warsaw, Poland). The pH was adjusted by gradually adding 0.1 mol/dm^3^ NaOH (titrant), using an automatic microburette (Dosimat 765, Metrohm, Herisau, Switzerland). The dependence of the σ_0_ value on the pH of the suspension was determined using the Titr_v3 computer program, which also controlled the titration process. These calculations were based on the difference in the volume of NaOH added to the suspension and to the supporting electrolyte solution to achieve the same pH value (Janusz, 1994), using the following equation (Equation 7):

$$\sigma_{0}=\frac{∆VcF}{mS}$$ (7)

where: *ΔV*–the difference in the volume of NaOH added to the suspension and to the supporting electrolyte solution to achieve the appropriate pH value [dm^3^]; *c*–NaOH concentration [mol/dm^3^], *F*–Faraday constant [C/mol], *m*–solid mass [g], *S*–solid surface area [m^2^/g].

The zeta potential (ζ) and aggregate size of activated biocarbon particles, both in the presence and absence of adsorbates, were evaluated using the procedure described below. Initially, a suspension of 200 cm^3^ volume was prepared, containing 200 ppm of tested polymer/s and/or metal ions, 0.001 mol/dm^3^ NaCl as a supporting electrolyte, and 0.03 g of the carbonaceous material. The suspension was subjected to ultrasonic treatment using an ultrasonic head (XL 2020; Misonix, Farmingdale, United States) for 3 min. Appropriate adsorbates were then introduced into the suspension. The obtained suspension was divided into smaller portions, and the pH of each portion was adjusted to a value ranging from 3.0 to 10.0 (±0.1), using 0.1 mol/dm^3^ HCl and NaOH solutions. pH adjustment was performed using a Φ360 pH meter (Beckman, Brea, United States). The zeta potential was determined using the laser Doppler electrophoresis technique with a Zetasizer Nano ZS (Malvern Instruments, Malvern, United Kingdom). This instrument measured the electrophoretic mobility of solid particles, both covered with adsorption layers and without them. The zeta potential (ζ) was then calculated from the electrophoretic mobility data using Henry’s equation (Equation 8): (Ohshima, 1994):

$$U_{e}=\frac{2\varepsilon_{0}\varepsilon \zeta }{3\eta }f\left(\kappa a\right)$$ (8)

where: *U*_*e*_–solids electrophoretic mobility [cm^2^/(V⋅s)], *ε*_*0*_ – the electric permittivity of a vacuum [F/m], *ε*–dielectric constant, *η*–viscosity of dispersive phase [Pa⋅s], *f(κa)* – Henry’s function.

The measurement was carried out 9 times and the error did not exceed 5%.

Additionally, the stability of the suspension was investigated by determining the average size of solid particles aggregates at pH 3, 6, and 9. In this case, the measurements were based on the static light scattering phenomenon. Each sample was measured three times and the error was less than 5%.

## Results and discussion

### Physicochemical characteristics of carbonaceous materials

Both obtained activated biocarbons have a mesoporous structure and well-developed specific surface area (Table 1). The material obtained from the mint herb residue shows significantly better textural parameters (surface area 666 m^2^/g), while the adsorbent derived from the nettle herb remains is characterized by a greater concentration of the functional groups on its surface (Table 2). Both of these parameters have a strong impact on the activated biocarbons adsorption capacity. The average pore size of both adsorbents is approximately 2 nm, which allows to classify these materials as mesoporous. The surface of examined solids is dominated by basic functional groups, the concentration of which for NE_AP material is about 14 times higher than acid groups. The points of zero charge (pzc) occur at pH 8.5 and 9.7 for nettle- and mint-based samples, respectively, which confirmed the alkaline properties of both adsorbents. On the other hand, the isoelectric points (iep) of activated biocarbons (iep) are located at pH below 3 for NE-AP activated biocarbon and at pH 3.5 for MT_AP one, which are significantly lower than the pzc values. It may be the result of partial overlapping of the electrical double layers (edl) formed on the walls of the pores, which is a phenomenon often observed for mesoporous materials (Skwarek et al., 2014; Szewczuk-Karpisz et al., 2021).

**TABLE 1:** Textural parameters of the activated biocarbons.

Adsorbent	Surface area [m^2^/g]		Pore volume [cm^3^/g]		Average pore size [nm]	Micropore contribution
	Total	Micropore	Total	Micropore		
NE_AP	368	248	0.21	0.10	2.26	0.47
MT_AP	666	535	0.32	0.21	1.91	0.66

**TABLE 2:** Acid-base properties of the activated biocarbons.

Adsorbent	Acidic groups concentration [mmol/g]	Basic groups concentration [mmol/g]	Total concentration [mmol/g]	pH_pzc_	pH_iep_
NE_AP	0.32	4.57	4.89	8.5	<3
MT_AP	0.63	1.56	2.19	9.7	3.5

**FIGURE 2:**
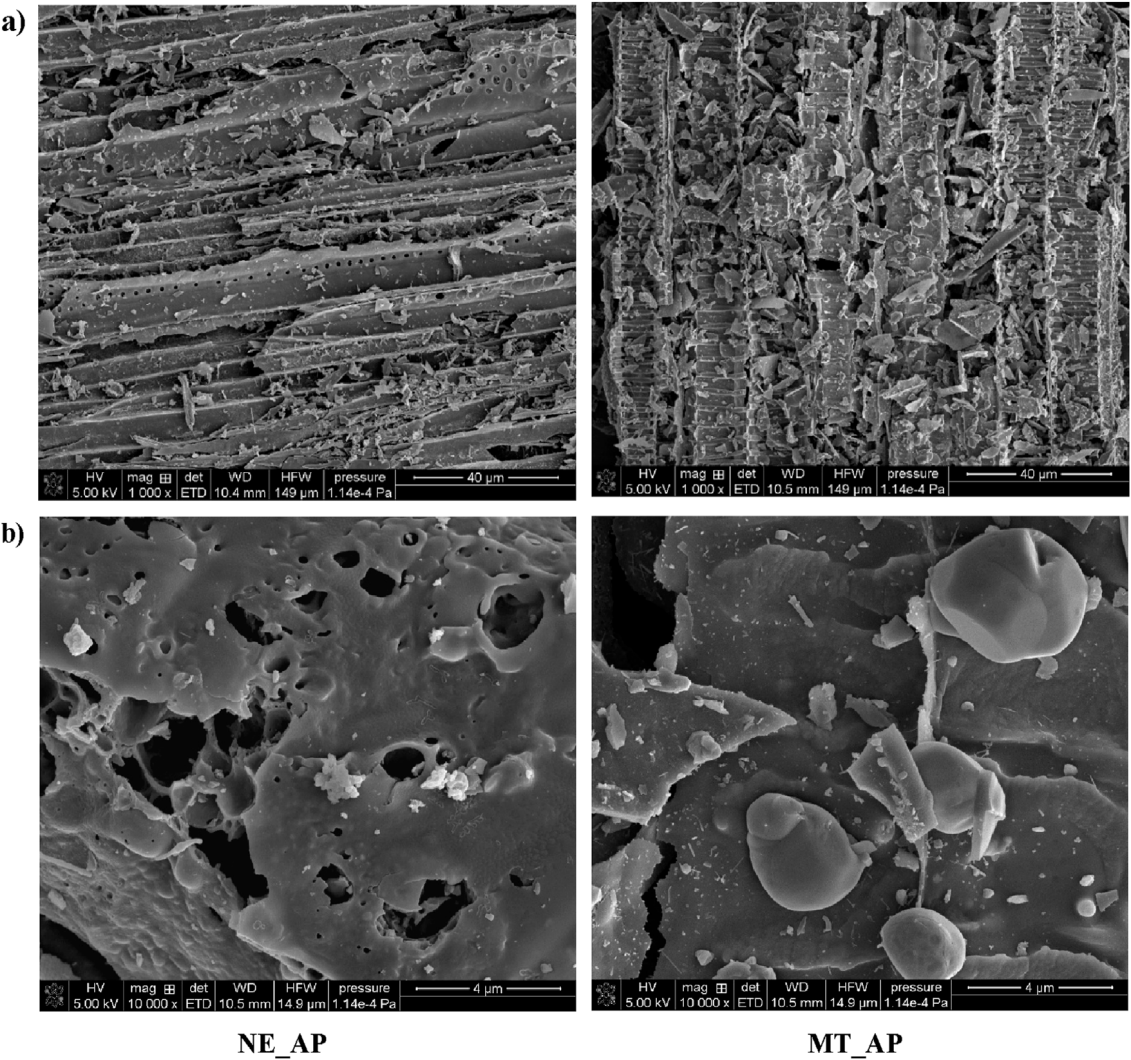
The SEM images of the activated biocarbons [**(a)**–zoom 1000x; **(b)**–zoom 10 000x].

Figure 2 presents the SEM images of the activated biocarbons. Despite the elongated shapes present in the structure of both materials, their surface layers differ significantly. The structure of NE_AP biocarbon is characterized by the presence of round holes and small pieces/particles on the surface. In the case of MT_AP material, the accumulation of smaller pieces on the surface is greater, beneath which a ribbed structure can be observed. These varied textural structures correspond to the activated biocarbons surface area, which is much greater in the case of the MT_AP sample.

**FIGURE 3:**
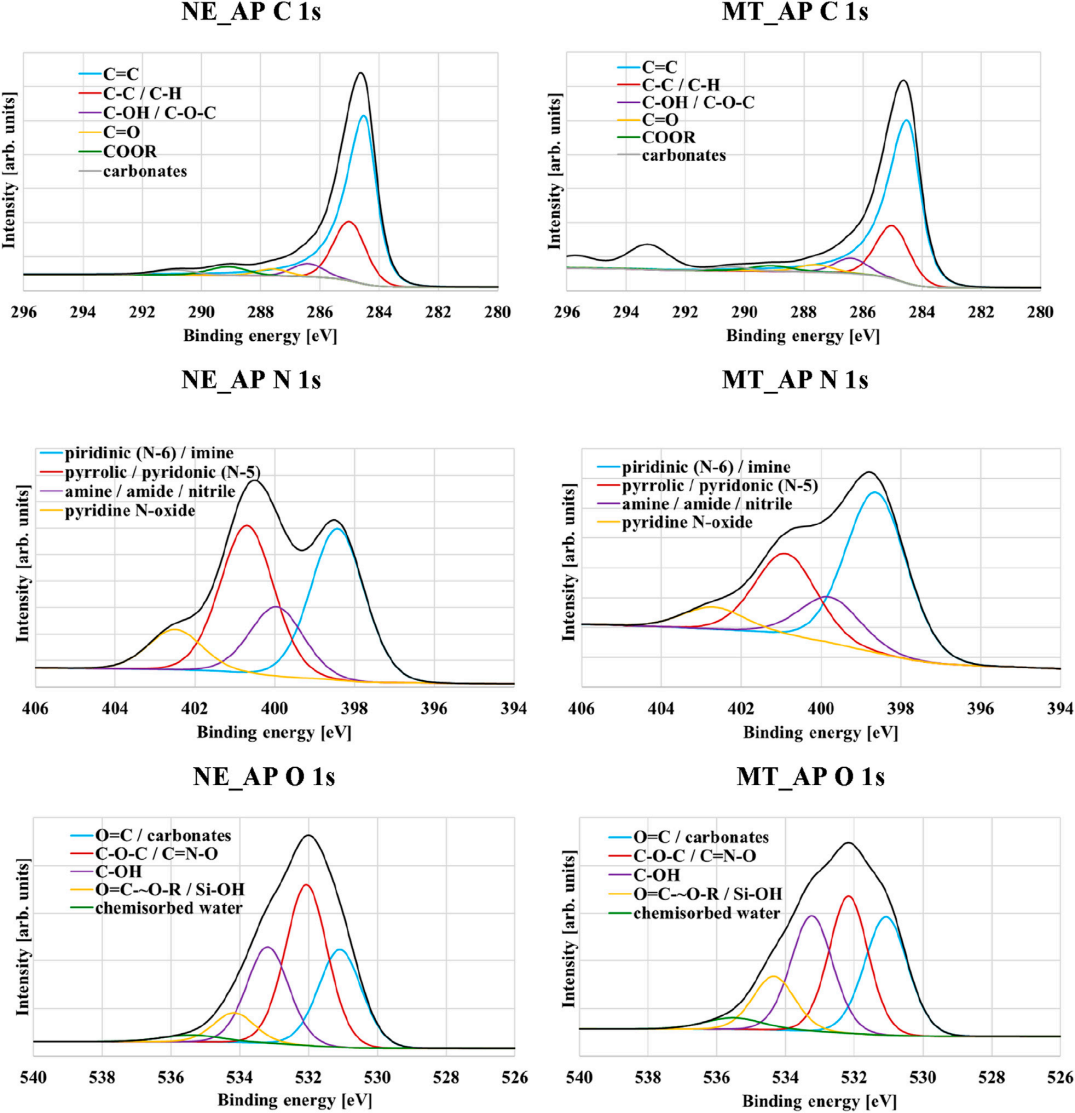
XPS spectra of C 1s, N 1s and O 1s regions for NE_AP and MT_AP activated biocarbons.

According to the data presented in Table 3 and in Figure 3, both materials are characterized by a high carbon content exceeding 75 at%. The structure of both activated biocarbons is clearly dominated by C=C groups. Furthermore, the contents of C-C, C-H, C-OH, C-O-C, C=O and carbonate species are similar in both solids. The second most abundant element is oxygen, with a concentration of about 13 at%. In the case of NE_AP activated biocarbon the contribution of carbonyl groups is the highest, while for the material obtained from mint herb residue, the contents of hydroxyl, carbonyl, and ether groups are at a similar level. In turn, the share of carboxyl groups is the lowest in both solids. The content of nitrogen does not exceed 2 at%, although the XPS tests confirm the presence of pyridinic, pyrrolic and amine groups. XPS analysis also revealed the presence of mineral admixtures in the structure of the obtained activated biocarbons, which is a fairly typical phenomenon for carbonaceous materials produced from plant-derived waste biomass.

**TABLE 3:** Chemical composition of the surface layer of activated biocarbons based on XPS analysis.

Element	Content of element [at%]	
	NE_AP	MT_AP
C	75.9	78.1
N	1.9	1.9
O	13.9	13.3
P	1.3	0.7
Cl	-	0.6
K	-	3.8
Ca	3.2	1.5
Mg	1.9	-
Si	1.8	-

The results of elemental analysis (summarized in Table 4) confirmed that carbon is the main component of the obtained materials. The high oxygen content is related to the large number of functional groups introduced as a result of partial gasification of the carbon matrix during heat treatment in the CO_2_ atmosphere. The obtained data also indicate a high ash content in the obtained activated biocarbons. This is a consequence of the presence of numerous mineral admixtures in the structure of the starting materials, which do not decompose during the activation process.

**TABLE 4:** Ash content and elemental composition of the activated biocarbons (wt%).

Sample	Ash	C^daf^	N^daf^	H^daf^	S^daf^	O^diff^
NE_AP	17.5	77.9	3.9	1.3	0.0	17.0
MT_AP	23.5	74.7	3.1	1.7	0.0	20.5

d^af^–dry-ash-free basis.

^diff^–calculated by difference.

Table 5 presents the results of the XRF analysis. No heavy metals were found in the composition of activated biocarbons, which proves that the obtained materials will not have a negative impact on the environment and their disposal should not pose any problems. The data show that, in addition to elements such as C, H, N, S, and O, both biocarbons also contain considerable amounts of phosphorus, calcium (particularly the sample derived from nettle) and potassium (especially the MT_AP material).

**TABLE 5:** Chemical composition of activated biocarbons based on XRF results.

Element	Content [mg/g]	
	NE_AP	MT_AP
P	0.075	0.119
S	0.012	0.024
Cl	0.003	0.124
K	0.005	0.333
Ca	0.320	0.399
Mn	-	0.003
Fe	0.002	0.005
Cu	-	0.005
Ga	0.001	-

### Adsorption capacity and regeneration efficiency of carbonaceous materials

**FIGURE 4:**
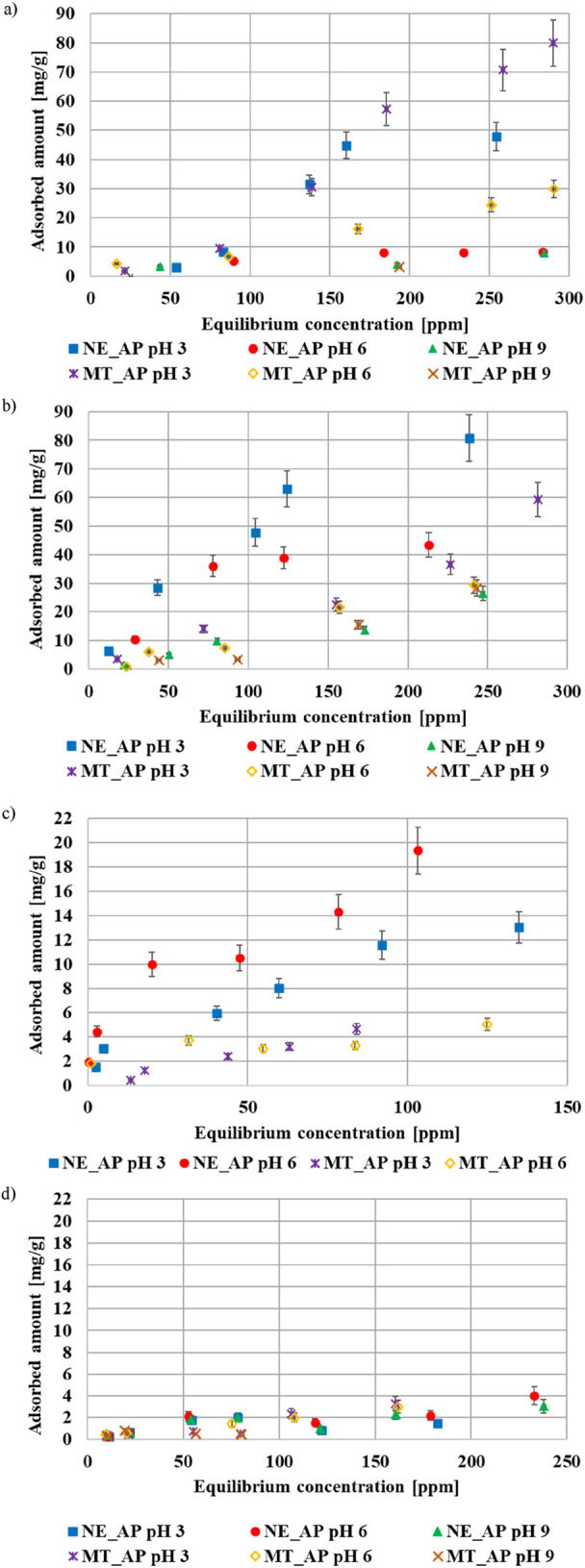
Adsorption isotherms of PEI **(a)**, PAA **(b)**, Cd(II) **(c)** and As(V) **(d)** on the activated biocarbons surface obtained at pH 3, 6, and 9.

The greatest adsorption of polyethyleneimine occurs at pH 3 (Figure 4a). Under these conditions, positively charged polymer chains are repulsed electrostatically by the positively charged adsorbent surface, forming an adsorption layer with perpendicular-orientated macromolecules. In turn, the increasing pH value causes the surface charge of the activated biocarbon to become negative, which results in the attractive interaction between the cationic polymer and adsorbent. The number of macromolecules adsorbed in parallel orientation increases, while the packing density of the adsorption layer decreases. Poly(acrylic acid) is also adsorbed in the greatest amount at pH 3 and its adsorption efficiency decreases with increasing pH. In this case, similarly to PEI, it is related to changes in the polymeric chain conformation (Figure 4b). At low pH values, most of the carboxyl groups is fully protonated and poly(acrylic acid) chains occur in a coiled conformation. The higher the pH is, the more PAA functional groups dissociate. This causes significant development of polymer chains, which create loosely packed adsorption layers and consequently lead to a reduction in the amount of adsorbed substance (Gęca et al., 2024a). The maximum adsorbed amounts of polymers observed in this study are higher than most values reported in the literature. The adsorption capacity of inorganic materials (such as metal oxides) towards ionic polymers does not exceed 30 mg/g (Alemdar et al., 2005; Chibowski et al., 2009; Liufu et al., 2005; Santhiya et a. 1999). In the case of activated carbons, the obtained results are comparable to our previous findings for carbonaceous materials derived from sage and lemon balm stalks via physical activation (Groszek et al., 2025).

The removal of cadmium(II) ions from an aqueous solution is less influenced by pH value compared to the polymers, but it is slightly more efficient at pH 6 (Figure 4c). In the case of As(V) ions, their adsorbed amounts are very low and similar in all systems (Figure 4d). The obtained isotherms data were fitted to theoretical models (Table 6). Both heavy metal ions’ adsorption is best described by Freundlich and Dubinin-Radushkevich models, which indicates that the adsorption process takes place inside the pores and leads to the formation of multilayer adsorbate films (Foo and Hameed, 2010). The maximum adsorbed amounts of cadmium ions reported in the literature often exceed 100 mg/g, but the adsorbents used in these studies were mainly synthesized using aggressive chemical reagents (Meng et al., 2024; Tan et al., 2022; Yin et al., 2023). The amounts of Cd(II) ions adsorbed on the surface of environmentally friendly adsorbents are comparable to the values obtained in the presented studies (Anwar et al., 2010; Farasati et al., 2016). The adsorption of As(V) ions has been studied primarily using novel, chemically modified inorganic materials, whose adsorption capacity ranges from 30 to 200 mg/g (Đolić et al., 2021; He et al., 2023; Wu et al., 2022). In contrast, the application of eco-friendly carbonaceous materials for As(V) ions removal has been investigated to a very limited extent.

**TABLE 6:** Calculated isotherms parameters of Cd(II) and As(V) ions adsorption on NE_AP activated biocarbon surface at pH 6.

Isotherm parameters		NE_AP	
		Cd(II)	As(V)
Experimental	q_exp_ [mg/g]	19.36	4.00
Langmuir	*q*_*m*_ [mg/g]	18.68	5.05
	*K*_*L*_ [dm^3^/mg]	0.0698	0.0064
	R^2^	0.8899	0.5424
Freundlich	*n*_*F*_	2.7480	1.4278
	*K*_*F*_ [mg/g·(mg/dm^3^)^1/n^]	0.0607	0.5333
	R^2^	0.9820	0.8665
Temkin	b_T_ [J/mol]	979	2673
	A_T_ [dm^3^/g]	4.0195	0.1118
	R^2^	0.8422	0.7374
Dubinin-Radushkevich	*β*	−3.0*10^−9^	−8.1*10^−9^
	ε [kJ/mol]	12.99	7.88
	*q*_*m*_ [mg/g]	37.96	18.03
	R^2^	0.9720	0.8810

**FIGURE 5:**
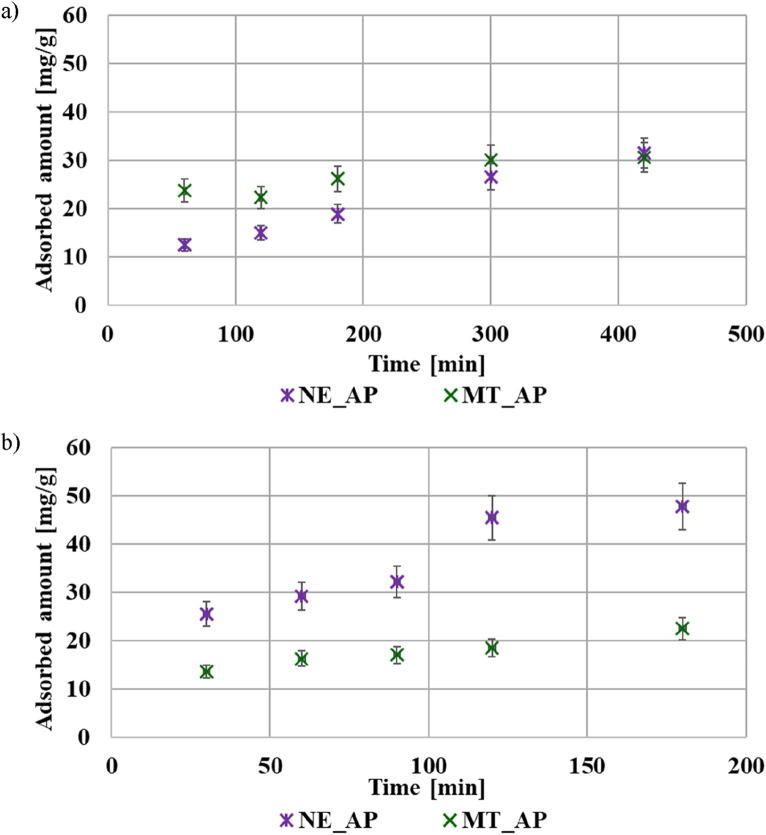
The adsorption kinetics of PEI **(a)** and PAA **(b)** on the surface of activated biocarbons at pH 3.

The adsorption kinetics results of the examined polymers are shown in Figure 5. The equilibrium state was reached after 6 h in the case of PEI and after 2 h in the case of PAA. This difference can be related to the size of both macromolecules at pH 3. The coiled poly(acrylic acid) molecules migrate faster through the bulk solution towards the adsorbent surface layer than the developed polyethyleneimine chains. The calculated theoretical parameters are listed in Table 7. Both polymers adsorption kinetics are better described by pseudo second-order model. This indicates that the polymer’s adsorption on the activated biocarbons surface involved mainly electron exchange between adsorbent and adsorbate (chemical adsorption mechanism). Due to the presence of functional groups–carboxyl in the case of PAA, and amine in the case of PEI, hydrogen bonds can be formed between the polymeric chains and the functional groups present on the activated biocarbons surface (Wiśniewska et al., 2021).

**TABLE 7:** Calculated kinetic parameters for the adsorption of PEI and PAA on the activated biocarbons surface at pH 3.

Kinetic parameters		NE_AP		MT_AP	
		PEI	PAA	PEI	PAA
Experimental	q_exp_ [mg/g]	31.45	47.79	30.54	22.45
Pseudo-first-order model	*q*_*e*_ [mg/g]	1.0140	1.0377	1.0130	1.0293
	*k*_*1*_ [1/min]	10.4164	1.6189	7.6639	8.5941
	*R* ^2^	0.8240	0.8917	0.9468	0.7916
Pseudo-second-order model	*q*_*e*_ [mg/g]	46.0829	76.3359	33.6700	25.6410
	*k*_*2*_ [g/(mg*min)]	0.00009	0.00013	0.00069	0.0011
	*R* ^2^	0.9300	0.8944	0.9909	0.9699

**FIGURE 6:**
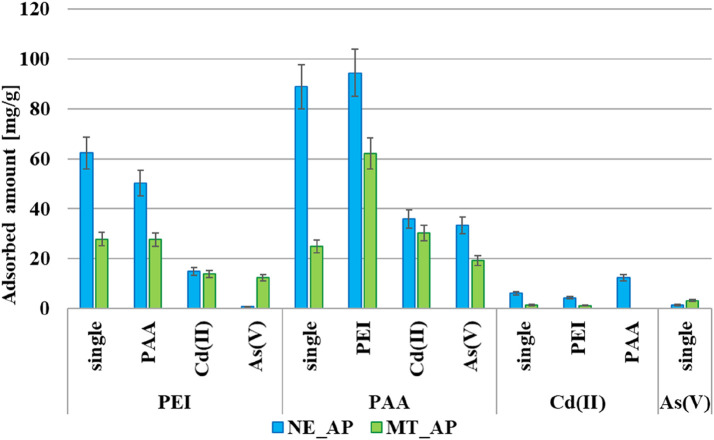
The adsorbed amounts of PAA, PEI, Cd(II) and As(V) on the surface of the activated biocarbons from single and binary solutions (C_0_ 200 ppm, pH 3).

According to the analysis of data presented in Figure 6, for most of the tested systems, activated biocarbon obtained from the nettle herb is a more effective adsorbent from both single and mixed adsorbate solutions. This confirms that the concentration of surface functional groups is of great importance for the adsorption capacity of carbonaceous material.

Poly(acrylic acid) and polyethyleneimine have a positive influence on their mutual adsorption (Santhiya et al., 1999). The adsorbed polymer chains can bind other macromolecules from the solution, forming multilayer adsorption structures (Gęca et al., 2024b). This effect is more noticeable in the case of PAA adsorption in the presence of PEI. Cationic polymer macromolecules are characterized by the larger linear dimensions at pH 3, which results in a greater number of amine functional groups that can interact with carboxyl groups of PAA. In the opposite situation, the coiled chains of the anionic polymer have fewer available functional groups, resulting in a smaller increase in PEI adsorption.

In solutions containing both the cationic polymer PEI and Cd(II) ions, the adsorbed amounts of each component from the binary mixture are lower than in the corresponding single-component systems. This behaviour is characteristic of cadmium cations in multicomponent systems. In solutions containing Cd(II) together with Pb(II)/Zn(II), the amount of adsorbed cadmium ions is reduced by 40%–80% compared to a single-component Cd(II) systems (Lee and Shin, 2021; Wang et al., 2022; Li et al., 2023). Both substances with the same ionic character compete for active sites, which negatively impacts their simultaneous removal from the aqueous phase. In a binary solution of poly(acrylic acid) and cadmium(II) ions, polymer-metal complexes are created, which depending on the type of adsorbent can be adsorbed on the activated biocarbons surface or remain in the solution. However, PAA is generally adsorbed in greater amounts from the binary solution than the Cd(II) cations. The presence of As(V) anions causes a decrease in the amounts of both polymers adsorbed. Moreover, the presence of the macromolecular compounds completely inhibits the adsorption of inorganic anions. This phenomenon can be attributed to the formation of complexes between arsenic(V) ions and organic substances, which rather tend to remain in the solution than adsorb on the surface (Gęca et al., 2024c). Conversely, it has been shown that the presence of small amounts of inorganic compounds can increase the amount of As(V) adsorbed on the surface of chemically modified adsorbents. These findings emphasize the need for more detailed studies on the removal mechanisms operating in multicomponent systems (Wei et al., 2021; Zeng et al., 2024).

**FIGURE 7:**
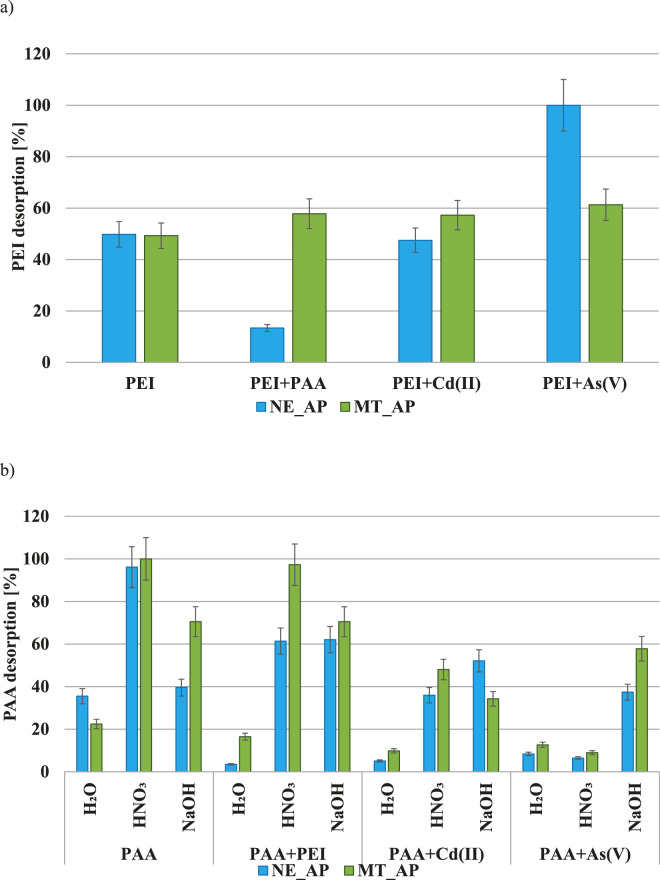
PEI **(a)** and PAA **(b)** desorption efficiency from the activated biocarbons surface with the usage of various desorption agents.

The desorption efficiency of the polymers is presented in Figure 7. Due to the impossibility of determining the concentration of polyethyleneimine in a solution with high ionic strength, in the case of this polymer only water was used for desorption tests. The most efficient desorption was observed in systems with As(V) ions, which can be related both to the small amount of adsorbed PEI and the weak binding of the polymer-metal complex to the adsorbent surface. The lowest desorption obtained in the NE_AP + PEI + PAA system indicates the strongest polyethyleneimine affinity to the surface of activated biocarbon derived from the nettle herb in the presence of poly(acrylic acid). In other systems the PEI desorption efficiency was about 50%.

In the case of PAA, H_2_O was the least effective desorption agent. HNO_3_ turned out the most effective substance in the single and PAA + PEI systems. In the presence of metal ions, NaOH allowed achieving the highest level of desorption. In general, the anionic polymer is better desorbed from the surface of MT_AP activated biocarbon, which proved that more than twice lower concentration of the adsorbent functional groups is crucial for the PAA removal from the aqueous solution.

**FIGURE 8:**
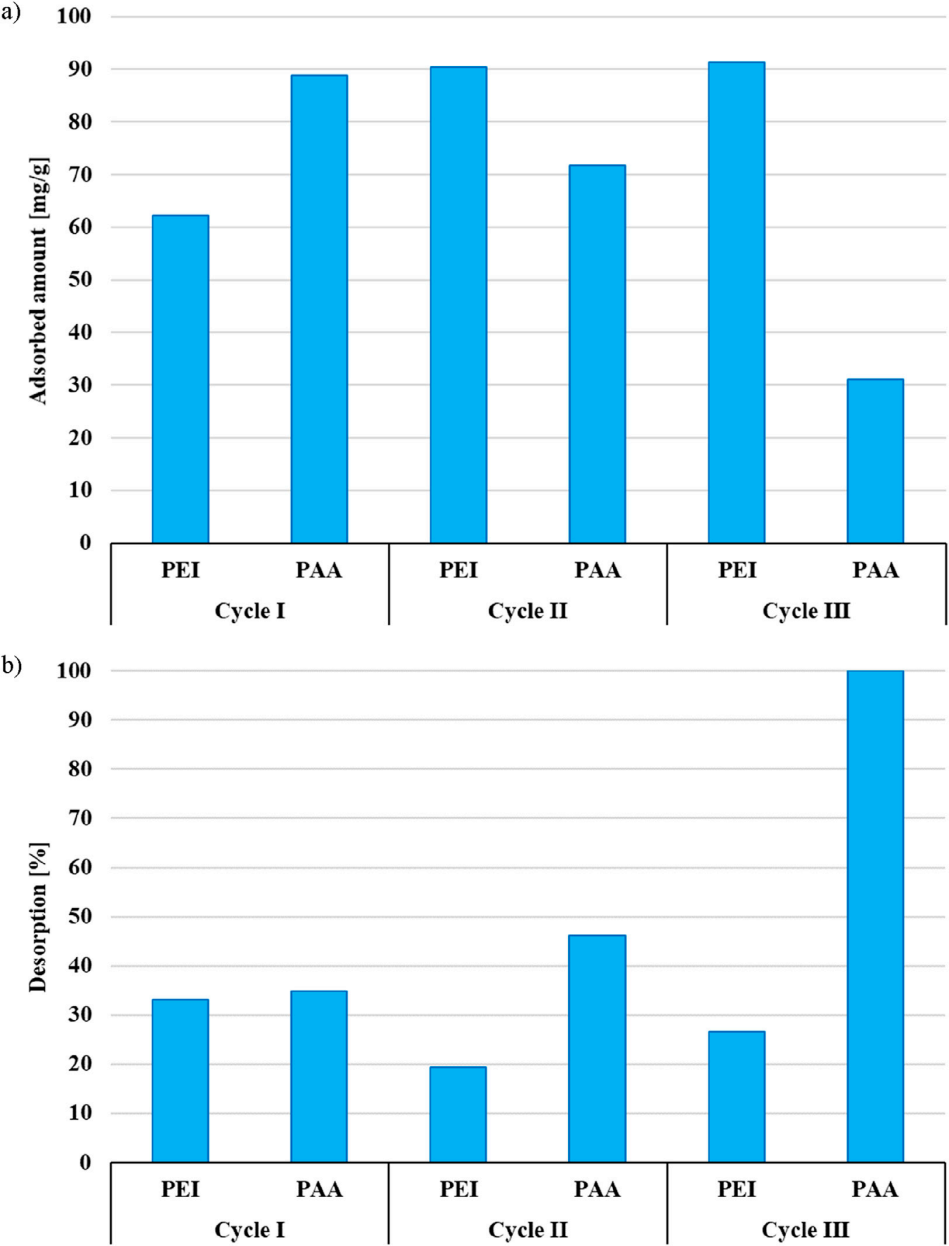
The adsorbed amount **(a)** and desorption efficiency **(b)** of polymers on/from the surface of NE_AP activated biocarbon in the adsorption-desorption cycles.

According to the data presented in Figure 8 the adsorbed amount of PEI increases with the increasing number of cycles. Due to the incomplete desorption (Figure 8b) of the cationic macromolecule and its perpendicular adsorption, the number of solid active sites increases. The permanently bonded PEI chains modify activated biocarbons’ surface, increasing their adsorption capacity. On the other hand, the PAA adsorbed amount decreases in the subsequent cycles. It is related to the adsorption of coiled polymeric chains in the solid pores, which remain not desorbing, blocking these sites for other PAA macromolecules.

### Electrokinetic properties and aggregation tendencies of aqueous suspensions of the carbonaceous materials

**FIGURE 9:**
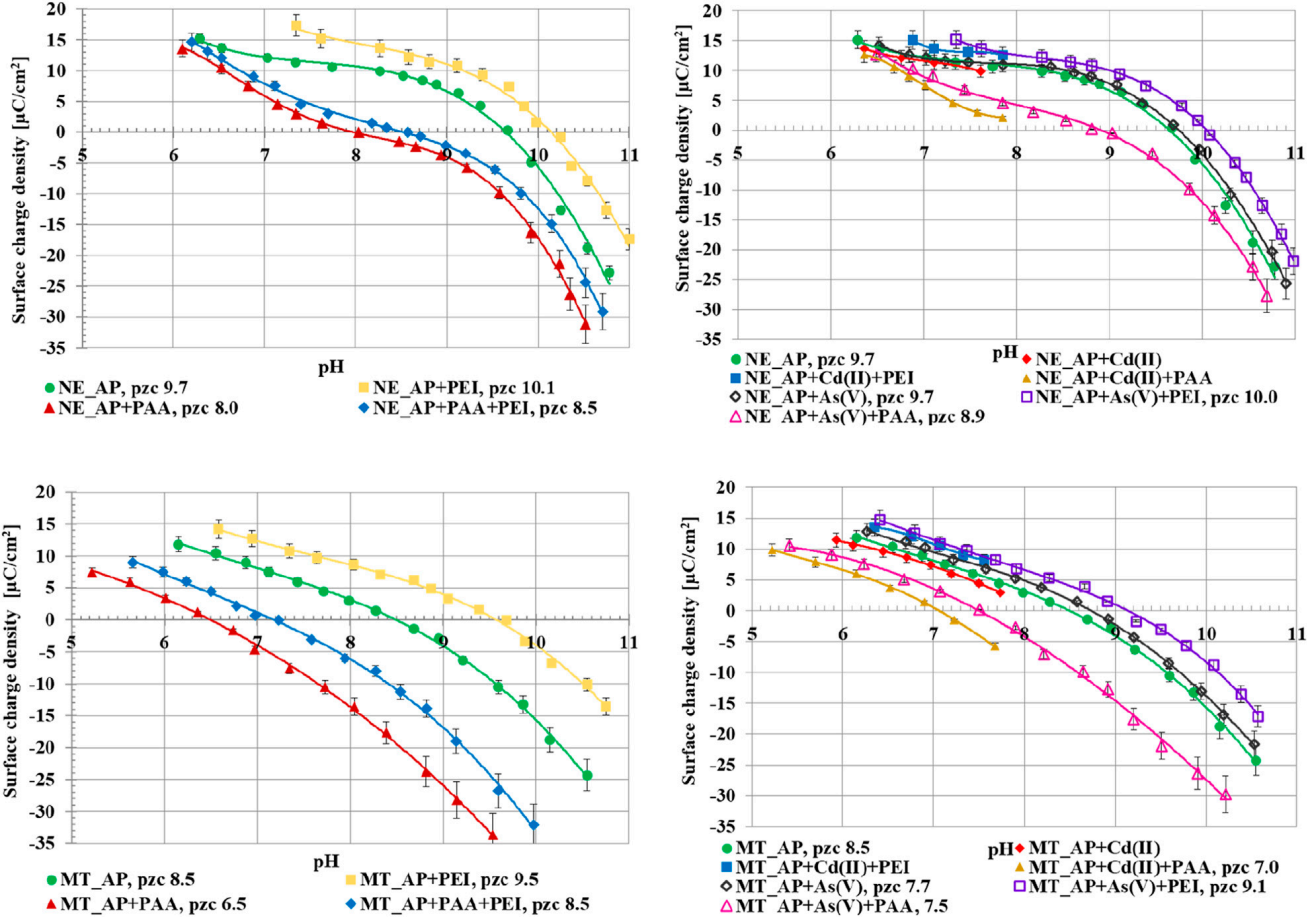
Surface charge density as a function of solution pH for NE_AP and MT_AP activated biocarbons particles dispersed in suspensions without as well as with single and binary adsorbates.

The dependencies of the surface charge density (σ_0_) of the activated biocarbons, for the systems without as well as with single and binary adsorbates, as the function of solution pH are presented in Figure 9. Polyethyleneimine causes an increase in activated biocarbons surface charge density value, whereas the presence of poly(acrylic acid) decreases this parameter value. In both cases, it is related to the fact that not all segments of the adsorbed macromolecules are directly bound to the adsorbent active sites. The functional groups are also present in the loop and tail structures, located in the by-surface area, which increase or decrease the σ_0_ parameter, according to the adsorbate chain charge (Wiśniewska and Nowicki, 2020). In the binary solution of two polymers, the intermediate value of the σ_0_ is observed. However, the predominant impact of PAA is noticeable, due to its greater adsorption in the studied system.

In the solution containing As(V) ions, a slight increase in the surface charge density is observed. The adsorbing arsenic anions induce the creation of an additional number of surface groups with a positive charge. On the other hand, the adsorption of Cd(II) cations favours formation of additional number of negatively charged sites on the activated biocarbons surface, resulting in a decrease of surface charge density. It is a typical behaviour observed for small inorganic ions (Fijałkowska et al., 2019). In the systems of ionic polymers and heavy metal ions, the effects accompanying macromolecules adsorption have a predominant impact on the σ_0_ value, due to their large sizes and significantly greater adsorbed amounts.

**FIGURE 10:**
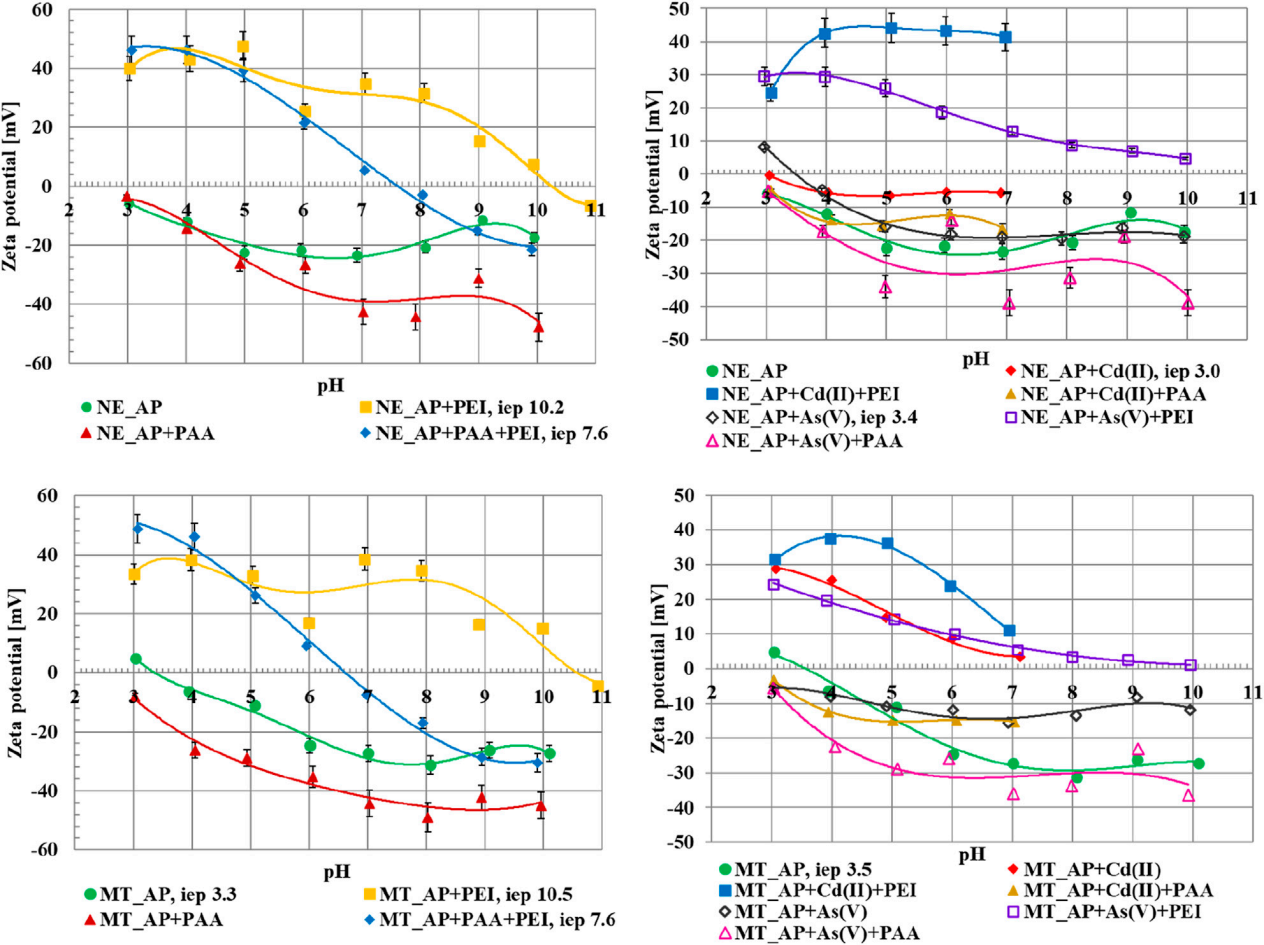
Zeta potential as a function of solution pH for NE_AP and MT_AP activated biocarbons particles dispersed in suspensions without as well as with single and binary adsorbates.

The changes of zeta potential (ζ) of the activated biocarbons particles dispersed in the systems without as well as with single and binary adsorbates as the function of solution pH are shown in Figure 10. Polyethyleneimine adsorption causes an increase in zeta in zeta potential value, which can be explained by the presence of polymeric chains with the positively charged amine groups in the slipping plane area. Anionic polymer binding leads to a decrease in the ζ value as a result of the analogous mechanism (Gęca et al., 2022). The zeta potential in the suspension containing both polymers increases at low pH values, at which the dissociation of the PEI chain is the greatest. The higher the pH value is, the greater the dissociation of carboxyl groups present in the PAA molecules is, and the resultant lowering of electrokinetic potential value is noted.

In the presence of Cd(II) ions the zeta potential value increases. The change of the ζ value to less negative after divalent cations (Cd^2+^) adsorption in comparison to monovalent supporting electrolyte ones (Na^+^) is a common effect, described in the literature before (Yalçınkaya and Güler, 2006; Yukselen-Aksoy and Kaya, 2011). Due to the small adsorbed amount of the As(V) ions, their influence on the zeta potential value is not very noticeable. In the system containing Cd(II) ions and polymers, an intermediate effect to their single solutions is observed, whereas the simultaneous presence of polymers and arsenic(V) ions results in obtaining dependencies similar to those observed for single PAA or PEI systems.

The aggregate sizes of activated biocarbons suspensions without as well as with single and binary adsorbates are included in Table 8. In single adsorbate systems, the adsorbent aggregate sizes increase in comparison to the suspensions without additives. The adsorption layers of organic/inorganic substances can cause the attraction between activated biocarbons particles resulting in suspension destabilization. The small metal ions neutralize the activated biocarbons’ surface charge which results in the coagulation of solid particles, whereas the polymeric chains in addition to the charge neutralization can also cause the bridging flocculation. Similar effects are observed in the binary systems containing As(V) ions, whereas the influence of Cd(II) ions in the presence of ionic polymers depends on the adsorbent type. On the other hand, the simultaneous presence of PEI and PAA has a stabilizing influence on the suspension. It can be explained by the interchain complexes creation which prevents the attraction between adsorbent particles (oppositely charged polymers neutralize each other’s charge).

**TABLE 8:** The size of aggregates formed by activated biocarbons particles in suspensions without as well as with single and binary adsorbates.

System	Aggregates size [nm]					
	NE_AP			MT_AP		
	pH 3	pH 6	pH 9	pH 3	pH 6	pH 9
Without adsorbates	2945	2411	1918	2040	2215	2229
PEI	3218	2115	2191	3137	2848	3809
PAA	2905	2177	2007	4290	2399	3016
Cd(II)	3829	11239	-	2401	8957	-
As(V)	10280	2580	4043	2572	3767	4275
PEI + PAA	475	348	421	729	737	694
PEI + Cd(II)	5287	2667	-	1979	2045	-
PAA + Cd(II)	2630	1215	-	3143	2750	-
PEI + As(V)	3180	3487	6101	3020	3571	4793
PAA + As(V)	2491	3017	4161	5029	2469	3873

## Conclusion

The plant-derived materials have been successfully used for the production of environmentally friendly carbonaceous adsorbents. The obtained activated biocarbons are characterized by moderately-developed surface area (368–666 m^2^/g) and high concentration of the functional groups on their surface (2.19–4.89 mmol/g). The occurrence of a point of zero charge at high pH values (8.5–9.7) and predomination of basic functional groups on their surface proved that both materials show basic properties. The activated biocarbon derived from nettle herb residue shows the highest adsorption capacity for both polyethyleneimine and poly(acrylic acid). The maximum adsorbed amounts of the tested substances were 79.98 mg/g for PEI, 80.81 mg/g for PAA, 19.36 mg/g for Cd(II), and 4.00 mg/g for As(V).

The examined polymers and heavy metal ions have a small influence on the determination of their concentration in the mixed solution, however, it is easy to deal with this impact by the application of calibration curves obtained from the solution containing interferent. It was proved that polymers have a positive influence on their mutual adsorption, in turn, the simultaneous removal of ionic macromolecules and heavy metal ions results in a decrease in their adsorbed amounts. The examined activated biocarbons can be successfully regenerated through desorption. The most efficient system for PEI desorption was NE_AP + PEI + As(V)+H_2_O and for PAA - NE_AP + PAA + HNO_3_, in which polymer desorption reached 100%. Reuse of regenerated carbonaceous materials is also possible, as indicated by adsorption cycle studies.

## Data Availability

The raw data supporting the conclusions of this article will be made available by the authors, without undue reservation.
